# Methamphetamine in the Midwestern United States: a call for advocacy and action

**DOI:** 10.3389/fpsyt.2026.1712541

**Published:** 2026-02-19

**Authors:** Alëna A. Balasanova, Michelle Davids

**Affiliations:** 1 Department of Psychiatry, University of Nebraska Medical Center, Omaha, NE, United States; 2 Department of Psychiatry, Broadlawns Medical Center, Des Moines, IA, United States

**Keywords:** advocacy, harm reduction, methamphetamine, Midwest, stimulant use disorder

## Introduction: epidemiology and context

The rates of stimulant-related overdose deaths, especially those involving non-prescription methamphetamine, have risen sharply across the United States, with some estimates suggesting a staggering 50-fold increase between 1999 and 2019 (1). Many of these deaths involve co-ingestion of high-potency synthetic opioids (HPSOs) such as fentanyl, which is increasingly being found in supplies of methamphetamine unbeknownst to the user (2). Individuals naïve to opioids who unwittingly use fentanyl along with methamphetamine are at greater risk of unintentional overdose. Although opioid-related mortality remains pervasive, aggressive expansion of evidence-based treatments such as the United States (U.S.) Food and Drug Administration (FDA) approved medications methadone, buprenorphine, and naltrexone led to a nearly 37% decrease in synthetic opioid-related overdose deaths in 2024 compared to 2023 (3). Provisional data also suggests a modest decrease in psychostimulant (including methamphetamine) overdose deaths during the same time period, falling to an estimated 29,456 deaths in 2024 from 37,096 in 2023 (3). Although the downward shift in drug-related overdose deaths is welcomed news, the overall increased acceleration in methamphetamine related overdose deaths in the last decade is significant and warrants increased attention.

The prevalence of specific substances is often regional, and historically methamphetamine has had a large presence in the geographically Midwest and Western regions of the United States, with stimulant-related overdose trends showing increased mortality from cocaine in Eastern states and psychostimulants such as methamphetamine in Western states (1). Of note, the terms amphetamine and methamphetamine are often not well differentiated in the research literature. A recent study noted that amphetamine-related International Classification of Diseases (ICD) codes do not discriminate between methamphetamine use, other unregulated amphetamine use, or non-medical use of prescription amphetamine medication, though evidence suggests that non-prescription methamphetamine is the predominant psychostimulant to which such codes refer (4). Accordingly, we use the term methamphetamine for the remainder of this editorial.

General hospital admissions due to non-fatal overdose or other sequelae of methamphetamine use increased by an estimated 270% from 2008–2015 with the highest rates of admissions concentrated along the Western USA (4). Methamphetamine-related psychiatric hospitalizations across the country increased by nearly 79% between 2015–2019 with the Midwestern region experiencing a 145% increase in psychiatric admissions (5). Notably, half of all methamphetamine-involved psychiatric hospitalization were due to psychosis (5). In addition to individual-level impacts, methamphetamine use influences the health of families and communities. While the coastal United States continues to grapple with an overdose crisis fueled by HPSOs, methamphetamine is the top unregulated substance used in the two Midwestern states of Iowa and Nebraska (6, 7).

Rates of methamphetamine use in Iowa have historically been high and continue to rise at a rate faster than the rest of the country. Over a third of Iowans entering substance use treatment report using methamphetamine and available services for methamphetamine use disorder (MUD) have limited efficacy with patients frequently having multiple unsuccessful short-term treatment episodes without sustained recovery (6). In recent years Iowa has continually seen a decline in successful treatment completion for MUD which is thought to be attributable to the rising prevalence of use (6).

In Nebraska, the attorney general has identified methamphetamine as the “number one” drug threat in the state (7). From January to July 2025, the Drug Enforcement Agency Omaha Division seized 138% more methamphetamine in Nebraska than in all of 2024 and local treatment providers identify that year after year, methamphetamine is the number one unregulated substance for which people seek substance use disorder care (8). Additionally, the Nebraska Foster Care Review Office reports that 18% of all foster care placements are due to parental methamphetamine use (9).

In spite of the large-scale impact of methamphetamine and high volume of patients with MUD, treatment options remain limited and large gaps in care remain. In rural states most affected by methamphetamine such as Iowa and Nebraska, resources are scarce and interventions that are known to be effective for reducing morbidity and mortality such as syringe service programs and fentanyl test strips are illegal. Additionally, unlike for opioid use disorder, there are not yet any FDA approved medications for treatment of methamphetamine use disorder. Studies of the off-label medications mirtazapine and the combination of bupropion plus extended-release naltrexone have shown a reduction in methamphetamine use among research participants though effect sizes were small and the research conditions were not generalizable to all settings and populations (10–12). In the real world, these medications are frequently overlooked as options for various reasons, including lack of patient adherence to daily medication and potential contraindications with patients’ other co-occurring medical conditions. Contingency management is an evidence-based non-pharmacological intervention with the largest body of literature to support its efficacy yet remains difficult to implement in many settings due to objective factors such as infrastructure and costs as well as the greater, subjective problem of stigma (13). Contingency management is not an available treatment modality in Nebraska or Iowa.

## State of affairs: policy-maker support

The sociopolitical landscape of individual states often influences healthcare-related state laws, including regulations around medical practice. This can impact healthcare professionals’ ability to provide evidence-based interventions for MUD and result in reduced access to care for patients. Allocating state resources for evidence-based interventions for MUD is often not prioritized. There are federal sources of financial support such as the State Opioid Response (SOR) grant, which allows funds to be used for developing and delivering evidence-based programs for MUD, though utilization of such grants for creation and implementation of evidence-based interventions for MUD vary considerably state to state (14).

For example, fentanyl test strips, which are a low-cost tool to identify the presence of fentanyl in one’s substance supply to prevent unintentional opioid overdose when methamphetamine is contaminated with fentanyl, are one of the interventions for which SOR funds can be utilized (15). However, fentanyl test strips are illegal in the state of Iowa. Under Iowa law, any person who knowingly or intentionally manufactures, delivers, sells or possesses drug paraphernalia has committed a misdemeanor crime (16). Drug-checking equipment for the presence of substances such as fentanyl are included in this definition of drug paraphernalia. Over the years, several bills have been introduced in the Iowa legislature to remove drug-checking equipment from the definition of drug paraphernalia, most recently House File 699 in 2025 (17). Unfortunately, none of the bills have advanced and the definitions and penalties remain the same at the time of this writing.

Fentanyl test strips do not meet the statutory definition of ‘drug paraphernalia’ in Nebraska and are thus technically legal (18). However, there is no state-wide distribution initiative and state health authorities have not utilized existing federal grant dollars for obtaining fentanyl test strips or written fentanyl test strips into grant funding requests. It is not illegal for Nebraskans to independently purchase fentanyl test strips out of pocket on the internet, however there is no systematic way of distributing or obtaining fentanyl test strips from a healthcare provider or office. The lack of state health authority support for this low-cost intervention proven to reduce unintentional overdose death is largely suspected to be due to stigma. There is an erroneous perception held by some policymakers that providing people with a way to reduce the harms of substance use sends the message that substance use is condoned and encouraged, and despite evidence of the contrary, policymakers fear this will lead to an increase in substance use, particularly among youth (19). In Iowa, the idea of harm reduction has been characterized as ‘creating a false sense of security’ and this justification has been cited as a reason for continuing the ban for certain harm reduction strategies (20).

As boots-on-the-ground clinicians working with patients with substance use disorders and other co-occurring psychiatric conditions, we are all too familiar with the lack of resources for treating patients with MUD and the stigmatizing beliefs held against these patients in our respective states of Nebraska and Iowa. We have personally engaged in and continue to support grass-roots advocacy efforts to improve the resources available for patients with MUD and to help shift public perception about MUD to that of a public health problem. We describe some of these efforts below.

## Discussion: the way forward

Where state policymaker support has stalled, citizen-led grassroots risk-mitigation efforts have proliferated. A recent example is a special vending machine installed outside the Polk County Health Department in Des Moines, IA (21). The vending machine, provided by the Family Planning Council of Iowa, offers at no cost to the consumer, a variety of harm reduction and public health supplies spanning from naloxone to needle cleaning kits to sharps disposal containers. The vending machine is available for consumers without the need for a prescription, identification, or registration. Local non-profit organizations have also engaged in charitable efforts to increase availability of naloxone, a medicine instrumental to reducing overdose deaths for individuals using methamphetamine laced with opioids or vice versa. For example, the non-profit group Steps of Hope has provided no-cost naloxone distribution boxes in over 20 counties throughout Iowa (22). These grassroots initiatives have been critically important for lowering barriers to obtaining harm-reduction supplies within Iowa communities.

Although non-profit and charitable organizations have come to be associated with grassroots efforts in the harm reduction space, the importance of individual physician advocacy cannot be discounted. In Nebraska, the strong advocacy of a single physician (the first author) through the state medical association propelled forward Legislative Bill 307 in 2023 (23). This bill sought to authorize local municipalities governed by city councils and county boards to permit syringe service programs in their jurisdictions if the locally elected leaders chose to do so; the bill also provided an exception to penalties related to drug paraphernalia under state law. The bill had bipartisan cosponsors and advanced handily in a nonpartisan fashion through three required rounds of debate in Nebraska’s unique Unicameral Legislature. This was a tremendous win for public health and for improving the lives of individuals with MUD (24). Alas, the bill was vetoed by the governor and did not become law (19). The justification provided in the official veto letter was that the bill would be “encouraging minors to abuse dangerous drugs,” and that syringe service programs do not reduce disease transmission and actually increase overdose deaths (19). The U.S. Centers for Disease Prevention refute these myths explicitly on their public-facing website, stating that over 30 years of research show that syringe service programs do not increase illegal drug use or crime and are associated with an estimated 50% reduction in HIV and hepatitis C incidence (25).

Even though the erroneous claims cited in the Governor’s veto letter have been disproven repeatedly by decades of data, the sentiments expressed capitalized on the pervasive stigma that has historically accompanied substance use in conservative regions of the country. The veto override vote failed with several of the legislators who previously voted in support flipping their votes, demonstrating how the sociopolitical landscape of a state can directly impact the health of the people living in it. Despite the veto of LB 307, the momentum gained from its initial passage has been sustained and efforts are underway to educate the recently elected slate of new policymakers about the importance of syringe service programs and to lay the foundation for legislation that may be introduced under a future administration.

Raising awareness about the need for increased resources for MUD treatment and advocating for policies to benefit patients living with this condition is an important part of what we do as front-line clinicians. Educating the next generation of medical learners regarding recognition and treatment of MUD and leveraging physician technical expertise to train the future healthcare workforce is another form of advocacy that must be encouraged and expanded. Both of us authors are heavily involved in medical education and training in our respective states and incorporate non-psychiatry learners on our addiction-treatment focused clinical services. Integrating healthcare professionals-in-training in this manner has allowed a larger footprint in the battle against stigma and helps promote improved identification and treatment when caring for patients who use methamphetamine.

As physicians, we take an oath to do no harm. The status quo in our respective states is doing harm to our patients by preventing the provision of evidence-based harm reduction interventions that we know to be effective. Inaction contributes to this harm. We conclude that both grassroots and individual healthcare professional-led advocacy and educational efforts are needed to encourage regulatory and statutory changes in state laws to increase the availability of evidence-based services for patients with MUD and to improve outcomes for our patients.
